# Organogermanium suppresses cell death due to oxidative stress in normal human dermal fibroblasts

**DOI:** 10.1038/s41598-019-49883-7

**Published:** 2019-09-20

**Authors:** Tomoya Takeda, Sota Doiyama, Junya Azumi, Yasuhiro Shimada, Yoshihiko Tokuji, Hiroaki Yamaguchi, Kosuke Nagata, Naoya Sakamoto, Hisashi Aso, Takashi Nakamura

**Affiliations:** 1 Asai Germanium Research Institute Co., Ltd., 3-131, Suzuranoka, Hakodate, Hokkaido 042-0958 Japan; 2 Department of Life and Food Sciences, Obihiro University of Agriculture and Veterinary Medicine, Nishi 2 Sen, Inada, Obihiro, Hokkaido 080-8555 Japan; 3 Department of Pharmaceutical Sciences, Tohoku University Hospital, 1-1, Seiryo, Aoba, Sendai, Miyagi 980-8574 Japan; 4 Present Address: Yamagata University Graduate School of Medical Science/Department of Pharmacy, Yamagata University Hospital, 2-2-2, Iidanishi, Yamagata, Yamagata 990-9585 Japan; 5 Department of Natural History Sciences, Hokkaido University, kita10jonishi, Kita, Sapporo, Hokkaido 060-0810 Japan; 6 Isotope Imaging Laboratory, Creative Research Institution, Hokkaido University, kita10jonishi, Kita, Sapporo, Hokkaido 060-0810 Japan; 7 Graduate School of Agricultural Science, Faculty of Agriculture, Tohoku University, 468-1, Aramaki aza, Aoba, Sendai, Miyagi 980-8578 Japan

**Keywords:** Cell death, Cellular imaging

## Abstract

Reactive oxygen species (ROS) are very harmful to dermal cells, and it is thus important to develop cosmetics that protect the skin from ROS and other stimuli. Repagermanium is a synthetic water-soluble organogermanium polymer, and in this study, we attempted to visualize the incorporation of germanium into normal human dermal fibroblasts (NHDFs) using isotope microscopy. In addition, the content of 3-(trihydroxygermyl)propanoic acid (THGP), a hydrolyzed monomer of repagermanium, in NHDFs was determined through liquid chromatography mass spectrometry (LC-MS/MS), and the dose-dependent incorporation of THGP was confirmed. We then evaluated the preventive effects of THGP against ROS-induced NHDF death and confirmed the observed preventive effects through gene profiling and expression analysis. The addition of 0.59–5.9 mM THGP reduced cell death resulting from ROS damage caused by the reaction between xanthine oxidase and hypoxanthine and the direct addition of H_2_O_2_. Furthermore, this study provides the first demonstration that the effect of THGP was not due to the direct scavenging of ROS, which indicates that the mechanism of THGP differs from that of general antioxidants, such as ascorbic acid. The gene profiling and expression analysis showed that THGP suppressed the expression of the nuclear receptor subfamily 4 group A member 2 (*NR4A2*) gene, which is related to cell death, and the interleukin 6 (*IL6*) and chemokine (C-X-C motif) ligand 2 (*CXCL2*) genes, which are related to the inflammatory response. Furthermore, the production of *IL6* induced by H_2_O_2_ was suppressed by the THGP treatment. Our data suggest that the preventive effect of THGP against ROS-induced cell death is not due to antioxidant enzymes or ROS scavenging.

## Introduction

Repagermanium, also known as Ge-132, is a water-soluble organogermanium compound and a polymer of 3-(trihydroxygermyl) propanoic acid (THGP; (HO)_3_GeCH_2_CH_2_COOH), and its safety has been confirmed by several toxicological studies^1–5^. In fact, repagermanium has recently been used as an ingredient in some foods and cosmetics in Japan and as a food supplement in the United States of America. Repagermanium was first developed for medical use^6^ and has been tested in pharmacological studies and clinical trials^7–13^. Repagermanium has the immunostimulatory properties of inducing interferon-γ and activating natural killer cells and macrophages^7,8^, and these effects result in tumor suppression^9,10^. Repagermanium has also been reported to alleviate cancer pain and reduce the use of morphine as a pain killer^11,12^. In addition, THGP, a hydrolytic monomer of repagermanium, has been reported to interact with some biological cis-diol compounds, such as adrenaline, ATP and nucleic acids^14,15^, and repagermanium exerts a curative effect on burn injuries^16^. Terminal cancer and burn injuries cause pain through the leakage of ATP from necrotic cells, and THGP might therefore be beneficial for pain control in patients with these diseases. Moreover, Matsumoto *et al*. recently reported that repagermanium promotes the rapid wound healing of injured skin in rats^17^. Therefore, skin care products containing repagermanium can be expected to have protective effects on dermal cells, but the absorption and incorporation of THGP into dermal cells has not been demonstrated.

Imaging studies have become popular and effective techniques for analyzing the localization of bioactive molecules. Unfortunately, THGP does not have a useful labeling moiety for visualization, such as a fluorescent tag, due to its simple structure. Isotope microscopy^18^ is useful for elemental analyses and has recently been used for elemental visualizations of biological samples, such as the localization of ^18^O of stable isotope-labeled RNA transfected into human lung adenocarcinoma epithelial cells and ^13^C of isotope-labeled glucose and ^15^N of isotope-labeled NH_4_NO_3_ in fungi and orchids^19,20^. Therefore, we investigated the localization of germanium in normal human dermal fibroblasts (NHDFs) through an isotope microscopy analysis.

Oxidative stress is one of the most harmful factors that attacks dermal cells. Various types of reactive oxygen species (ROS) disturb the functions and/or viability of skin cells^21^. Ultraviolet (UV) radiation in sunlight causes the formation of ROS in skin^22^, and the DNA damage induced by ROS causes dermal cells to become cancerous^23^. In addition, ROS produced by UV light also cause wrinkles, melanin spots, and other age-related skin conditions^24^. Therefore, the control of ROS generation in dermal cells is a very important issue for maintaining healthy and beautiful skin. Antioxidants, such as ascorbic acid (AA), are used in cosmetic products to reduce the formation of ROS in dermal cells^25^. ROS are generated in excessive amounts in burned and injured tissues^26^, and we previously described that THGP has a curative effect on burn injuries^16^. Therefore, the protective effect of THGP in skin tissue might depend on the direct or indirect control of ROS.

In this study, we first examined the incorporation and localization of THGP in cultured fibroblast cells through an isotope microscopy analysis. We then evaluated the preventive effect of THGP against ROS-induced cell death, and to elucidate the mechanism through which THGP prevents cell death, we subsequently performed a transcriptome analysis. The results obtained in this study reveal that the suppressive effect of THGP on ROS-induced cell death differs from that of general antioxidants, which directly scavenge ROS.

## Results and Disccusion

### Comparison of the material and molecular structure of Asai manufacturing and commercial repagermanium

**Figure 1:**
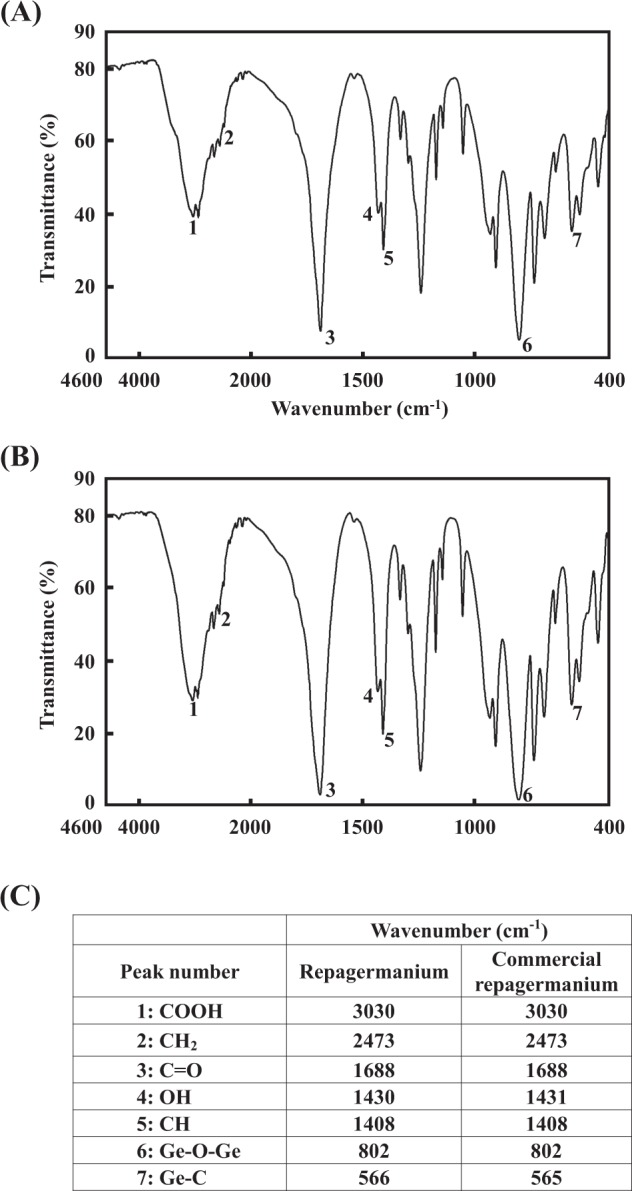
Material structure of Asai manufacturing and commercial repagermanium. Bis(2-carboxyethylgermanium(IV) sesquioxide) purchased from a common chemical vendor is referred to as commercial repagemanium. The infrared absorption spectra of repagermanium was measured via the KBr method using FTIR. Absorption wavelengths of Asai manufacturing (**A**) and commercial repagermanium (**B**). The measuring wavenumber range was set to 4600–400 cm^−1^. (**C**) Characteristic peak wavenumber of repagermanium.

**Figure 2:**
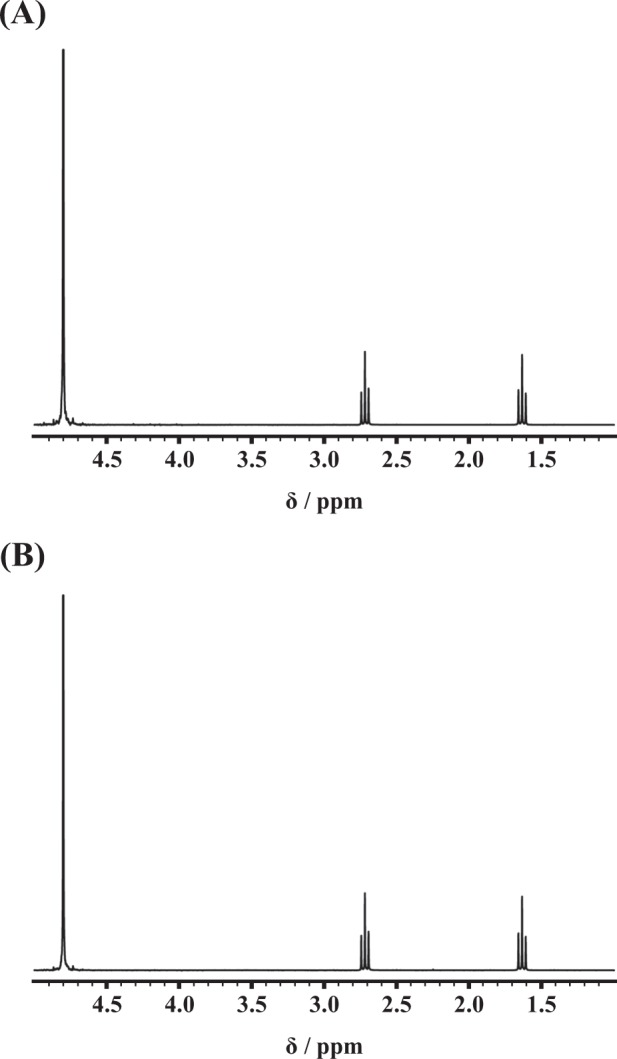
Molecular structure of Asai manufacturing and commercial THGP. Bis (2-carboxyethylgermanium(IV) sesquioxide) purchased from a common chemical vendor is referred to as commercial repagemanium. The molecular structure of the two repagermaniums was measured using NMR spectroscopy. The solution contained Asai manufacturing and commercial THGP, and all solutes were dissolved (20 mM each) in D_2_O. The ^1^H NMR spectra were measured at 300 MHz. ^1^H NMR spectra of Asai manufacturing (**A**) and commercial THGP (**B**).

Bis(2-carboxyethylgermanium(IV) sesquioxide) was purchased from a common chemical vendor and is referred to as commercial repagemanium. We compared differences in the material structure between Asai manufacturing and commercial repagermanium via Fourier transform infrared spectrophotometer (FTIR). The absorption wavelengths of Asai manufacturing and commercial repagermanium are shown in Fig. 1A,B, respectively, and the characteristic peak of repagermanium is shown in Fig. 1C. Compared with the FTIR data on repagermanium previously reported by Nakamura *et al*.^14^, the unique strong absorption wavelengths at 1686 cm^−1^ (peak 3) and 797 cm^−1^ (peak 5) originated from C = O and Ge-O-Ge in the Asai manufacturing repagermanium (Fig. 1A,C), and similar peaks were also observed in commercial repagermanium (Fig. 1B,C). Furthermore, other peaks characterizing repagermanium also coincided with that of the two repagermaniums (Fig. 1C). Because the two repagermaniums did not show an impurity peak and the shapes of the peaks matched completely (Fig. 1A,B), the material structures of the two repagermaniums are identical. Next, we examined the molecular structure of the two repagermaniums by nuclear magnetic resonance (NMR) spectroscopy, and the ^1^H NMR spectra are shown in Fig. 2A,B. Repagermanium is hydrolyzed to a THGP monomer when solubilized in water. Recently, Shimada *et al*. showed that the ^1^H NMR spectra of THGP have unique two proton triplet signals^15^, and these signals were observed in the THGP that solubilized each repagermanium, and the shape and chemical shift of the signals were completely identical (Fig. 2A,B). Therefore, there is no difference in the molecular structure of the two THGPs.

### Evaluation of the incorporation of THGP into NHDFs

**Figure 3:**
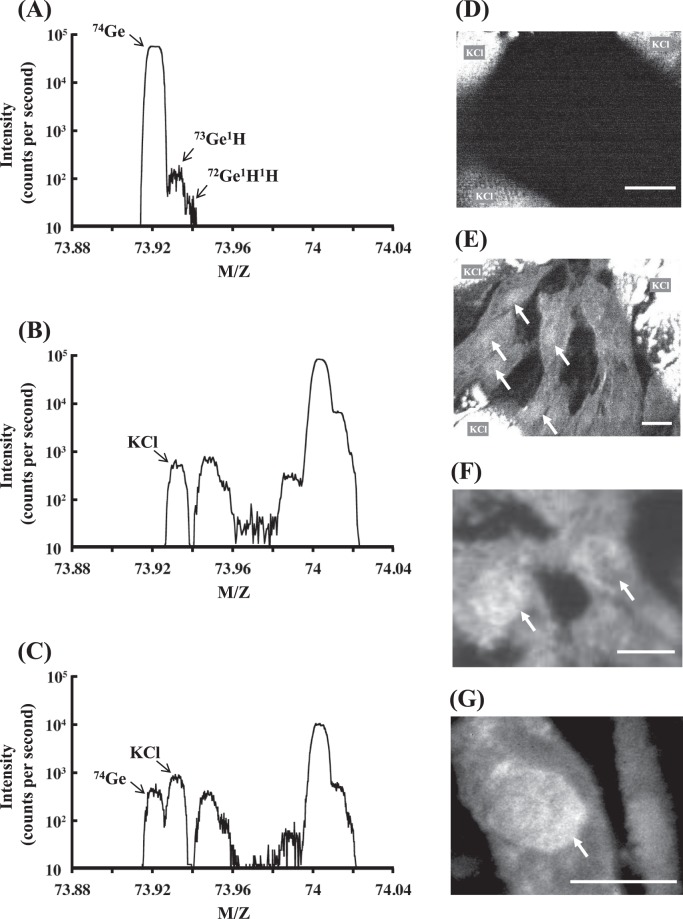
Mass spectra and germanium isotope images of NHDFs. NHDFs were cultured with or without 10 mM THGP on silicon wafers. Mass spectra of metallic Ge ingot (**A**) and NHDFs cultured in medium that were not treated (**B**) or treated (**C**) with THGP. (**D** and **E**) Isotope images of ^74^Ge in NHDFs not treated and treated with THGP. The bright regions at the upper right, left and lower left corners of the ^74^Ge images in (**D**) and (**E**) show interference by ^39^K^35^Cl. (**F** and **G**) Isotope images extracted from ^74^Ge images of THGP-treated cells. The white arrows indicate Ge-enriched regions. The scale bars represent 25 μm.

**Figure 4:**
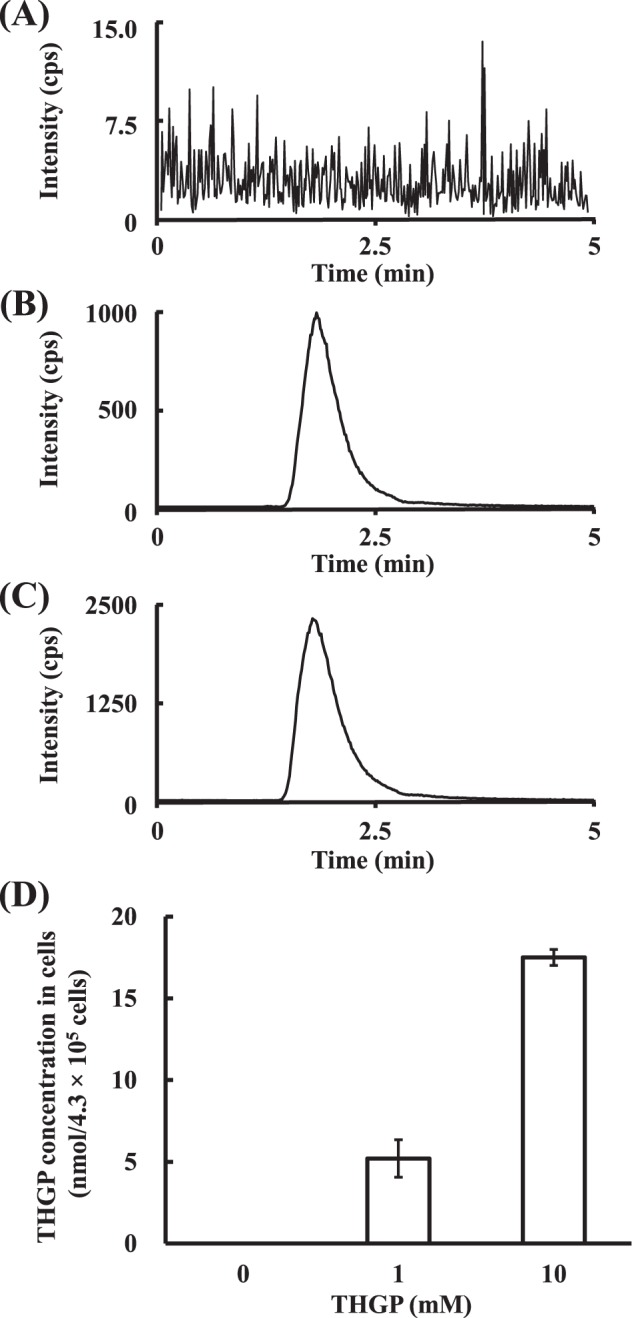
Identification of incorporated THGP in NHDFs. NHDFs were cultured for 24 h with THGP at concentrations of 0, 1 and 10 mM, and the incorporation of THGP in NHDFs was quantified using LC-MS/MS. Chromatogram of NHDF samples treated with 0 (**A**), 0.1 (**B**) and 1 mM THGP (**C**). (**D**) Concentration of THGP in NHDFs calculated from the chromatograms. The data are shown as the means, and the bars indicate the SEMs (n = 3).

Repagermanium (the polymer of THGP), which is effective against skin damage^16,17^, is expected to exert some effect on dermal cells, but its incorporation into dermal cells has not been reported. Therefore, we studied the incorporation of THGP using two different mass spectrometry-based methods. First, we attempted to visualize the distribution of germanium in the cells through isotope microscopy. Figure 3 shows the mass spectra near M/Z = 74 and the isotope images of ^74^Ge. As demonstrated by the mass spectra of metallic Ge ingot, which is shown in Fig. 3A, the main peak appears at M/Z = 73.92100, which indicates the presence of ^74^Ge with small peaks corresponding to ^73^Ge^1^H and ^72^Ge^1^H^1^H. The mass spectral analysis revealed the absence of a signal due to ^74^Ge in the cells that were not treated with THGP (Fig. 3B), whereas a clear peak for ^74^Ge was observed next to ^39^K^35^Cl in the THGP-treated cells (Fig. 3C). An exit slit of the isotope microscope can be narrowed to cut the interference of ^39^K^35^Cl except for ^74^Ge. The interferences of ^39^K^35^Cl appeared as bright regions at the corners in the untreated cells, similar to previous results (Fig. 3D)^27^. In contrast, Fig. 3E clearly shows the distribution of ^74^Ge in the THGP-treated cells except for the interference of ^39^K^35^Cl at the corners, similar to Fig. 3D. Figure 3F, G present large-scale images of ^74^Ge in the THGP-treated cells and reveal that ^74^Ge was more concentrated in the nucleus than in the cytosol. We subsequently identified the incorporation of THGP through LC-MS/MS analysis. The THGP content in the NHDFs is shown in Fig. 4. The chromatograms of the samples treated with each THGP concentration are shown in Fig. 4A–C, and the THGP concentration calculated from the data is shown in Fig. 4D. In fact, as shown in Fig. 4D, THGP was incorporated into the cells in a dose-dependent manner. Thus, we confirmed the incorporation of THGP into NHDFs by two different methods, and both analyses support its incorporation. In the imaging analysis, we only searched for only germanium atoms, whereas in the identification study, we screened for the entire THGP molecule.

### Effects of THGP on the prevention of NHDF death caused by oxidative stress

**Figure 5:**
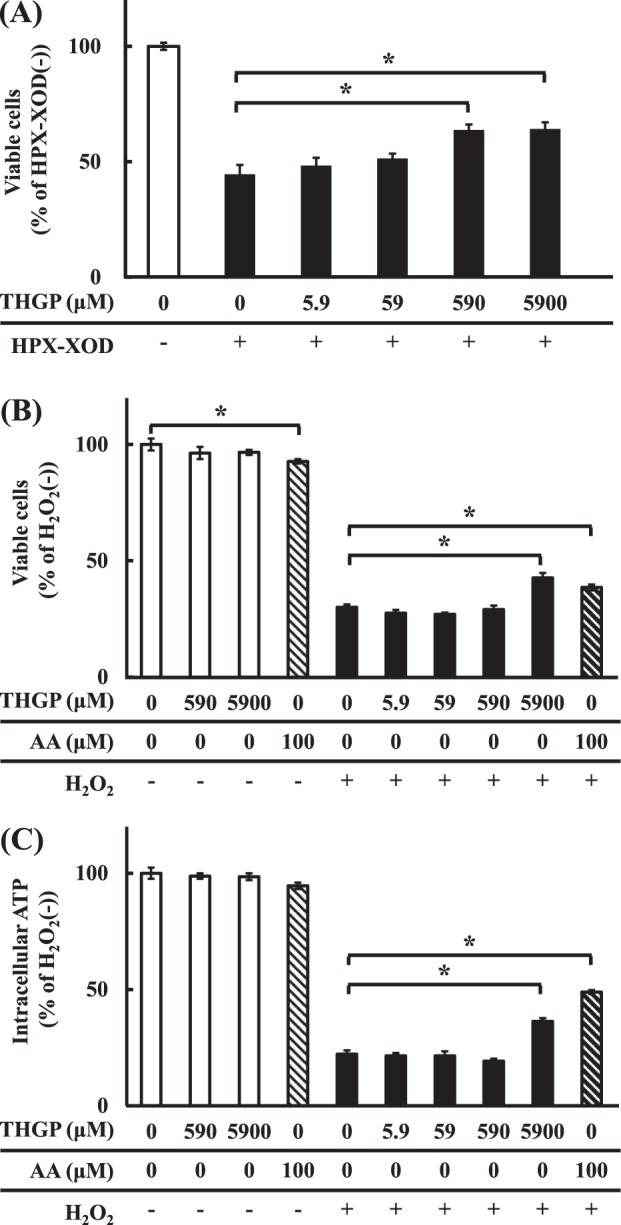
Effect of THGP on the viability and intracellular ATP of NHDFs exposed to oxidative stress for 1.5 h. The viability and intracellular ATP of NHDFs were measured via the MTS method and a luciferin-luciferase reaction, respectively. (**A**) NHDFs were cultured with or without added THGP at a concentration of 0, 5.9, 59, 590 or 5900 µM, and oxidative stress was induced by the addition of 10 U/l XOD and 0.3 mM HPX. (**B** and **C**) NHDFs were cultured with or without THGP or ascorbic acid (AA). THGP was added at a concentration of 0, 5.9, 59, 590 or 5900 µM, and AA was added at a concentration of 100 µM. Oxidative stress was induced by the addition of 5 mM H_2_O_2_. The data are shown as the means, and the bars indicate the SEMs (n = 8). The asterisk indicates significant differences at p < 0.05. (**A**) Viable cells after exposure to HPX-XOD stress. (**B**) Viable cells after exposure to H_2_O_2_ stress. (**C**) Intracellular ATP levels after exposure to H_2_O_2_ stress.

We then investigated the effect of THGP on the viability of NHDFs under conditions of oxidative stress, such as the presence of hydroperoxide generated by xanthine oxidase (XOD) and hypoxanthine (HPX). Figure 5A shows the preventive effect of THGP against NHDF death caused by oxidative stress. The percentage of viable NHDFs was significantly decreased by hydroperoxide stress (44.6%) compared with that of control cells (HPX-XOD(−)). At concentrations of 590 and 5900 µM, THGP exerted significant inhibitory effects on cell death compared with the HPX-XOD-treated control cells (HPX-XOD(+)). The percentages of viable NHDFs after treatment with 5.9, 59, 590 and 5900 µM THGP were 48.4, 51.4, 63.8 and 64.2% of the percentages found for the untreated control cells, respectively. The addition of THGP protected the cells from oxidative stress-induced death in a dose-dependent manner. We examined the effect of THGP on cell death caused by direct exposure to hydrogen peroxide (H_2_O_2_), and as shown in Fig. 5B. THGP did not change the percentage of viable cells at a concentration of 590 and 5900 µM (96.3%, 96.6%), whereas the addition of AA at 100 µM slightly but significantly reduced (92.7%) the percentage of viable cells without oxidative stress. Under oxidative stress, the percentage of viable NHDFs was significantly decreased (30%) compared with that found in the H_2_O_2_(−) cells, but THGP and AA exerted significant inhibitory effects on cell death at concentrations of 5900 (42.6%) and 100 µM (38.5%), respectively, compared with those found in the H_2_O_2_(+) cells. However, in this experiment using H_2_O_2_, THGP did not inhibit cell death at a concentration of 0.59 mM, but it is possible that the direct addition of H_2_O_2_ (30.0%) resulted in stronger oxidative stress than the addition of HPX-XOD (44.6%). In the enzymatic study, H_2_O_2_ was generated slowly starting from low concentration, whereas a constant H_2_O_2_ content was obtained with the direct method. Moreover, we attempted to measure the amount of intracellular ATP in order to the monitoring the percentage of viable cells in comparison with that obtained with the MTS method under similar oxidative stress conditions, and the results are shown in Fig. 5C. Under H_2_O_2_ oxidative stress, the intracellular ATP amount was significantly decreased (22.3%) compared with that found for the H_2_O_2_(−) cells; however, the addition of THGP and the addition of AA at concentrations of 5900 (36.4%) and 100 µM (49.0%), respectively, significantly increased the intracellular ATP content. In contrast, 590 and 5900 µM THGP and 100 µM AA did not significantly change the amount of intracellular ATP in the absence of oxidative stress (98.8, 98.5 and 94.6%).

In addition, Asai and commercial THGP did not differ in their ability to alleviate cell death (Supplementary Figure S1).

### Evaluation of NHDF death after 24 h of exposure to hydrogen peroxide stress through propidium iodide (PI)/Hoechst 3334215 (Hoechst) staining

**Figure 6:**
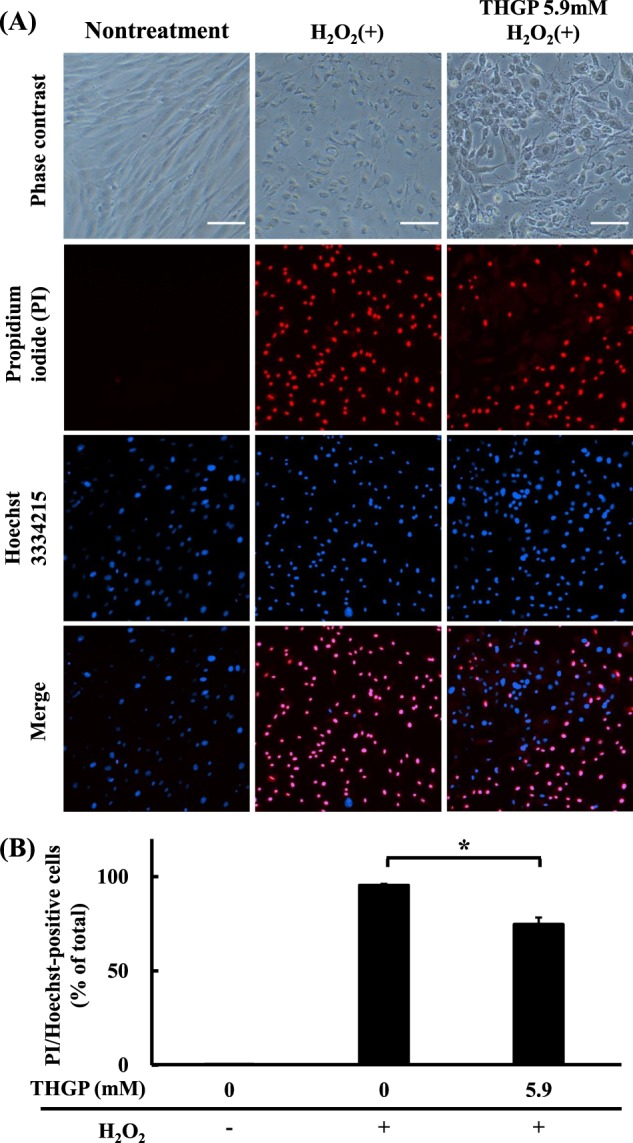
Effect of THGP on cell death of NHDFs under oxidative stress for 24 h. The death of NHDFs was confirmed via propidium iodide (PI) and Hoechst 33342 staining. NHDFs were cultured with or without 5.9 mM THGP, and oxidative stress was induced by the addition of 5 mM H_2_O_2_ for 24 h. The cells were then stained with PI/Hoechst 33342. (**A**) The PI/Hoechst 33342 dyes showed normal and dead cells. The images were detected by a fluorescence microscope at × 100 magnification. The scale bars represent 100 μm. The PI-positive cells are shown in red, and the Hoechst-positive cells are shown in blue. Therefore, the double-positive cells are indicated in magenta. (**B**) The PI-and-Hoechst-positive cells were counted as dead cells. Eight fields of 2 mm^2^ in area were randomly counted for the control and treated cells. The bars indicate the SEMs (n = 6, 8), and the asterisk indicates significant differences at p < 0.05.

We subsequently investigated the inhibitory effect of THGP on cell death caused by exposure to H_2_O_2_ oxidative stress for 24 h through PI and Hoechst staining. Figure 6 showed the microscopy observations: the PI/Hoechst staining data are shown in Fig. 6A, and the cell death percentages calculated based on the ratios of the number of PI-stained cells to the Hoechst-stained cells are shown in Fig. 6B. Although oxidative stress increased the percentage of PI-and-Hoechst-positive cells to 95.4%, the addition of THGP decreased the percentage to 74.6%. The results confirmed that THGP also significantly suppresses cell death due to prolonged oxidative stress and indicate that THGP exerts an inhibitory effect on oxidative stress-induced cell death. It has been reported that THGP suppresses cell death caused by oxidative stress^28^, and Wada *et al*. found that THGP (Ge-132) exerts a cell proliferative effect by increasing the intracellular ATP content. However, because NHDFs did not display increased intracellular ATP after THGP treatment in the absence of oxidative stress (Fig. 5C), we obtained a different response to THGP treatment, and this contradictory finding might be different due to the different cell type (or cell lines) used in the study. Therefore, we hypothesized that the inhibitory effect of THGP on cell death caused by oxidative stress occurs through another mechanism in NHDFs. In the past, our research group performed *in vivo* experiments that revealed that the oral intake of dietary repagermanium increases the levels of antioxidative compounds;^29^ therefore, THGP might regulate the antioxidative activity of antioxidant molecules and/or enzymatic reactions related to oxidative stress.

### Effect of the addition of THGP on catalase and superoxide dismutase (SOD) activities and amount of intracellular ROS in NHDFs under oxidative stress

**Figure 7:**
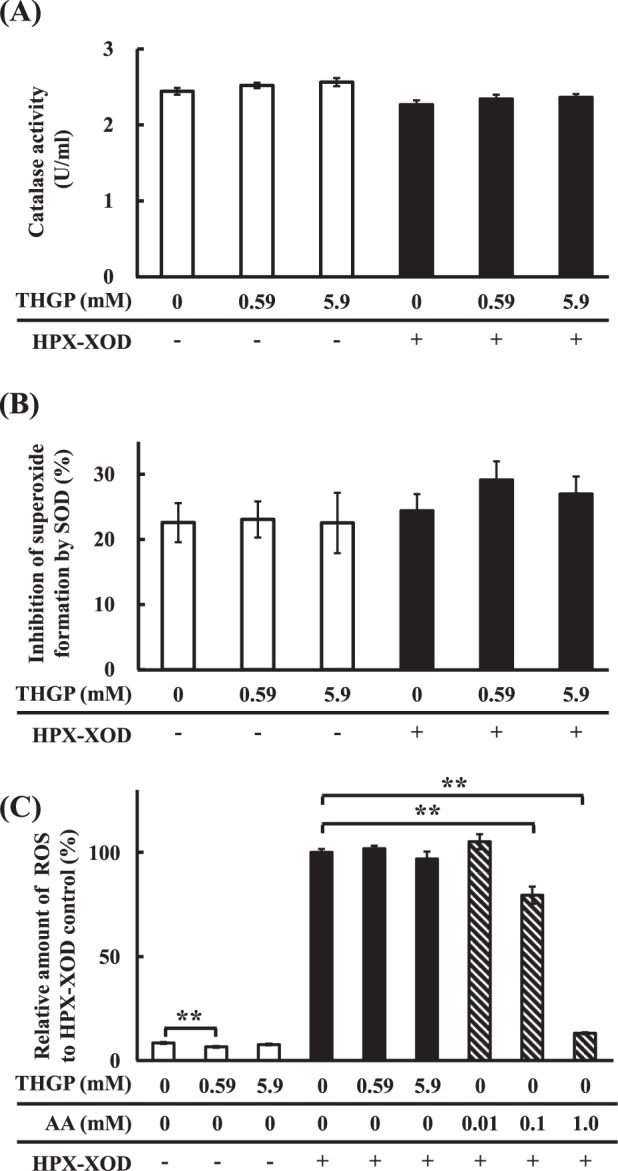
Effect of THGP on catalase and SOD activities and intracellular ROS in NHDFs. NHDFs were cultured with THGP at a concentration of 0, 0.59 or 5.9 mM or ascorbic acid (AA) at 0.01, 0.1 or 1.0 mM. Oxidative stress was induced by the addition of 10 U/l XOD and 0.3 mM HPX. The data are shown as the means, and the bars indicate the SEMs (n = 4, 6 or 8). Double asterisk indicates significant differences at p < 0.01. (**A**) Catalase activity. (**B**) SOD activity. (**C**) Intracellular ROS.

We then evaluated the effect of the addition of THGP on the activities of catalase and SOD, an enzyme that reduces ROS, under conditions of oxidative stress. We hypothesized that the addition of THGP would enhance the activity of catalase or SOD in NHDFs, and we thus attempted to confirm the likelihood that the ROS level was reduced by catalase and/or SOD. The effects of THGP addition on catalase and SOD activities are shown in Fig. 7A,B. As shown, no significant change in catalase activity was obtained after the addition of THGP, which indicated that THGP did not affect the enzymatic activity of catalase. SOD activity was measured in comparison with the activity of water, and the results showed that SOD activity in the NHDFs under conditions of oxidative stress was slightly increased by the addition of THGP, but this increase was not statistically significant. Tezuka *et al*. recently revealed that THGP regulates the activities of some redox-related enzymes, including catalase, in the liver homogenate of monkeys^30^. However, the present study revealed that THGP did not affect the activities of catalase and SOD. However, we noticed differences between the single cell line (of skin) and whole cell homogenates of tissue (liver), and detailed studies are required to further elucidate these mechanisms. The effect of THGP and AA on the amount of intracellular ROS is then shown in Fig. 7C. Although the amount of ROS in the cells increased due to oxidative stress, the suppressive effect of THGP was not observed. However, the intracellular ROS level in the normal state was significantly decreased by the addition of 0.59 mM THGP, although the slight decrease (1.9% lower than that obtained with HPX-XOD (−)) is not considered a reduction that suppresses oxidative stress-induced cell death. In contrast, AA at concentrations of 0.1 (79.4%) and 1.0 mM (13.2%) attenuated the amount of intracellular ROS in the presence of HPX-XOD compared with that obtained with HPX-XOD (+) cells (Fig. 7C). It is well known that AA itself exhibits antioxidant capacity and radical scavenging activity^31,32^. Therefore, we hypothesized that AA suppresses cell death by attenuating ROS in a dose-dependent manner. The present study revealed that the suppressive effect of THGP on cell death under oxidative stress is not caused by ROS scavenging by other well-known antioxidants, such as AA, polyphenol and carotenoids^33,34^. Although it has also been reported that THGP (Ge-132) itself has no antioxidative activity^35^, the intake of THGP (Ge-132) induces the antioxidative ability in the living body^29^. The present results are similar to those obtained in a previous *in vivo* study, which induced antioxidative effects through the oral administration of THGP (Ge-132). However, in this cell-based study, THGP did not induce increases in the contents of bilirubin and its metabolites, which indicates that it does not serve as an antioxidant; therefore, another mechanism must be responsible for the observed effect.

Furthermore, compared with the THGP of Asai, the commercial THGP did not affect intracellular ROS (Supplementary Figure S2).

### Effect of THGP addition on the gene expression profile and the protein expression of NHDFs under oxidative stress

**Figure 8:**
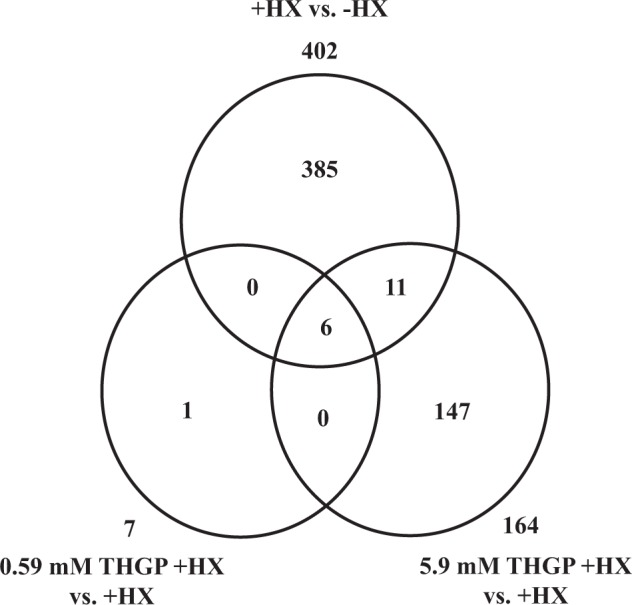
Comparison of genes whose expression levels were altered by THGP. The gene expression levels were compared among the -HPX-XOD (−HX), +HPX-XOD (+HX), 0.59 mM THGP + HX, and THGP 5.9 mM + HX cells. The differentially expressed genes were defined as genes that were up- or downregulated by more than 2-fold. The numbers in the Venn diagram show the differentially expressed genes.

A recent comprehensive gene expression profiling study uncovered the functions of THGP in the mouse liver^36^. In fact, the use of a DNA microarray to analyze gene expression in the liver of a mouse fed a 0.05% repagermanium-containing diet for one day detected many genes involved in immune reactions, such as the FC receptor, which suggested that repagermanium enhances the defense response of living organisms. Thus, we used a DNA microarray to evaluate the effects of THGP on NHDFs under oxidative stress and reveal the mechanism underlying the protective effect of THGP on ROS-induced cell death. The genes whose expression level was up- or downregulated by more than 2-fold between the different treatment groups were regarded as differentially expressed genes and used in further analysis. The expression of 402 genes was changed by ROS generated by HPX-XOD. Specifically, supplementation with 5.9 mM THGP up- or downregulated the expression levels of 17 genes by more than 2-fold after compared with the level obtained for the HPX-XOD(+) (+HX) group (Fig. 8). Six of the 17 genes were up- and downregulated by 5.9 mM THGP + HX and 0.59 mM THGP + HX (Table 1). The 17 identified genes were then analyzed using the DAVID bioinformatics tool according to the Gene Ontology (GO) biological function annotations. In total, 16 GO terms obtained for the 17 genes that were altered by THGP had a p-value < 0.05 (Table 2). Based on these GO terms, THGP exerts an effect on cell migration and response to hormones under oxidative stress. Moreover, the expression of the genes involved in the “response to oxygen-containing compound (GO:1901700)” was also affected by THGP. Because the cells were exposed to hydrogen peroxide in the presence of HPX-XOD, changes in the response to an oxygen compound stimulus due to THGP might have occurred.

**Table 1:** Effect of THGP treatment on gene expression in NHDFs under oxidative stress.

Probe ID	Gene Symbol	Public ID	Gene Name	Fold changes in gene expression		
				+HX vs −HX	0.59 mM THGP vs +HX	5.9 mM THGP vs +HX
204622_x_at	NR4A2	NM_006186	nuclear receptor subfamily 4, group A, member 2	3.84	0.10	0.12
216248_s_at	NR4A2	S77154	nuclear receptor subfamily 4, group A, member 2	4.00	0.13	0.13
204621_s_at	NR4A2	AI935096	nuclear receptor subfamily 4, group A, member 2	3.57	0.13	0.13
205207_at	IL6	NM_000600	interleukin 6	3.01	0.44	0.36
209774_x_at	CXCL2	M57731	chemokine (C-X-C motif) ligand 2	5.85	0.42	0.39
219228_at	ZNF331	NM_018555	zinc finger protein 331	5.14	0.37	0.41
202340_x_at	NR4A1	NM_002135	nuclear receptor subfamily 4, group A, member 1	2.26	0.54	0.48
209324_s_at	RGS16	BF304996	regulator of G-protein signaling 16	8.62	0.70	0.48
220987_s_at	AKIP1 NUAK2	NM_030952	A kinase (PRKA) interacting protein 1 NUAK family, SNF1-like kinase, 2	4.33	0.68	0.50
205925_s_at	RAB3B	NM_002867	RAB3B, member RAS oncogene family	0.33	1.90	2.16
220253_s_at	LRP12	NM_013437	Low-density lipoprotein receptor-related protein 12	0.47	1.62	2.18
215092_s_at	NFAT5	AJ005683	nuclear factor of activated T-cells 5, tonicity-responsive	0.23	1.59	2.41
220342_x_at	EDEM3	NM_017992	ER degradation enhancer, mannosidase alpha-like 3	0.33	1.19	2.59
211380_s_at	PRKG1	D45864	protein kinase, cGMP-dependent, type I	0.46	1.62	2.60
220484_at	MCOLN3	NM_018298	mucolipin 3	0.48	1.31	2.77
201856_s_at	ZFR	BC000376	zinc finger RNA-binding protein	0.47	1.99	2.97
211090_s_at	PRPF4B	Z25435	pre-mRNA processing factor 4B	0.38	1.29	5.01

**Table 2:** Enriched GO terms for genes identified from the NHDFs exposed to oxidative stressed and treated with THGP.

Gene Ontology (GO) term	Genes altered by 5.9 mM THGP	P-value	Genes						
GO:0045444 fat cell differentiation	3	0.010	IL6	NR4A1	NR4A2				
GO:0016477 cell migration	5	0.011	IL6	NR4A1	NR4A2	CXCL2	PRKG1		
GO:0071383 cellular response to steroid hormone stimulus	3	0.015	IL6	NR4A1	NR4A2				
GO:0060326 cell chemotaxis	3	0.016	IL6	NR4A1		CXCL2			
GO:0048870 cell motility	5	0.016	IL6	NR4A1	NR4A2	CXCL2	PRKG1		
GO:0051674 localization of cell	5	0.016	IL6	NR4A1	NR4A2	CXCL2	PRKG1		
GO:0071375 cellular response to peptide hormone stimulus	3	0.021	IL6	NR4A1	NR4A2				
GO:1901700 response to oxygen-containing compound	5	0.022	IL6	NR4A1	NR4A2	CXCL2		EDEM3	
GO:1901653 cellular response to peptide	3	0.024	IL6	NR4A1	NR4A2				
GO:0040011 locomotion	5	0.026	IL6	NR4A1	NR4A2	CXCL2	PRKG1		
GO:0033993 response to lipid	4	0.027	IL6	NR4A1	NR4A2	CXCL2			
GO:0048545 response to steroid hormone	3	0.034	IL6	NR4A1	NR4A2				
GO:0043434 response to peptide hormone	3	0.039	IL6	NR4A1	NR4A2				
GO:0006928 movement of cell or subcellular component	5	0.043	IL6	NR4A1	NR4A2	CXCL2	PRKG1		
GO:0045944 positive regulation of transcription from RNA polymerase II promoter	4	0.043	IL6	NR4A1	NR4A2				NFAT5
GO:1901652 response to peptide	3	0.046	IL6	NR4A1	NR4A2				

**Figure 9:**
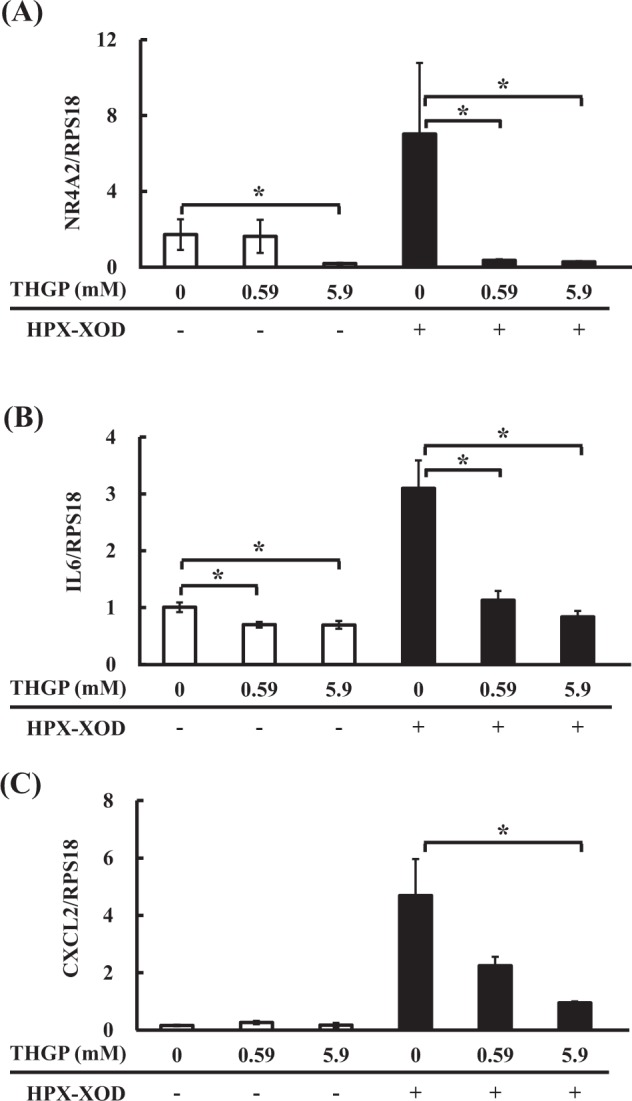
Effect of THGP on the expression of NR4A2 (**A**), IL6 (**B**), CXCL2 (**C**) in NHDFs. The expression levels of the NR4A2, IL6 and CXCL2 genes were measured by RT-qPCR and are shown relative to the expression of the RPS18 reference gene. THGP was added at a concentration of 0, 0.59 or 5.9 mM, and oxidative stress was induced by the addition of 10 U/l XOD and 0.3 mM HPX. The data are shown as the means, and the bars indicate the SEMs (n = 5 or 6). The asterisk indicates significant differences at p < 0.05.

Nuclear receptor subfamily 4 group A member 2 (NR4A2), interleukin 6 (IL6) and chemokine (C-X-C motif) ligand 2 (CXCL2) are commonly involved in several of the affected GO biological processes. The expression levels of these genes were confirmed by RT-qPCR. NR4A2 gene expression was significantly downregulated by 0.59 mM THGP + HX (0.36 ± 0.05) and 5.9 mM THGP + HX (0.28 ± 0.03) relative to that in the + HX group (7.02 ± 3.75) (Fig. 9A). Furthermore, similar to the gene expression fluctuations, intracellular NR4A2 protein expression tended to increase under oxidative stress and decrease under the THGP treatment (Supplementary Figure S3). Watanabe *et al*. recently reported that NR4A2 induces cell death and found that NR4A2 migrates out from the nucleus through the apoptosis signal-regulating kinase 1 (ASK1)-p38 pathway activated by oxidative stress and acquires a necrosis-inducing function^37^. Furthermore, Shi *et al*. suggested that NR4A2 is induced via the ROS-activated NFκB pathway and promotes autophagy-dependent apoptosis^38^. It is possible that the cell death-suppressing effect of THGP under conditions of oxidative stress is the result of the suppression of cell death induction by NR4A2. It has been reported that THGP treatment activates the apoptosis defense mechanism, which is primarily driven by the KEAP1-associated anti-apoptotic pathway^39^. In this experiment, the expression of genes encoding components of antiapoptotic pathways such as Keap-1, caspase and Bax, as reported by Kim *et al*., did not change, which might be due to the difference between metabolism-generated oxidative stress and exogenous oxidative stress. However, THGP might be involved in the control of cell death, possibly NR4A2-mediated cell death. The cytoplasmic increase and localization of NR4A2 protein was reported to induce cell death;^37,38^ thus, it is important to investigate the relationship between protein localization and quantity in the future to elucidate the cell death suppression mechanism of THGP.

**Figure 10:**
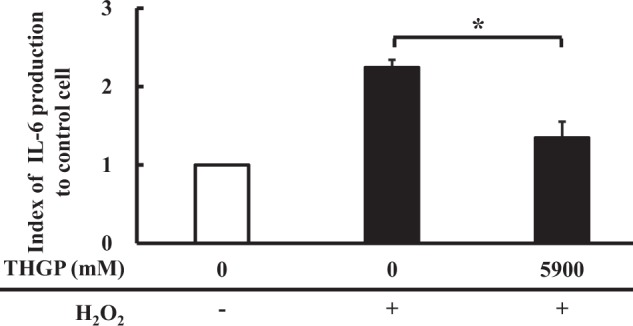
Evaluation of the production of IL6 of NHDFs. NHDFs were treated with 0.5 mM H_2_O_2_ in the medium with or without THGP. After 1.5 h, the culture supernatants were collected. The contents of IL6 were measured and corrected by the viable cell rate. The data are shown as the means, and the bars indicate the SEMs (n = 8). The asterisk indicates significant differences at p < 0.05.

ROS are known as a trigger of inflammatory responses and can cause tissue damage via inflammation^40,41^. IL6 is a well-known cytokine induced by an inflammatory reaction against stress, such as UV radiation^42^ and ROS^43^, and is therefore called an inflammatory cytokine. In this study, the IL6 gene expression levels were significantly upregulated by HPX-XOD (3.10 ± 0.49) and downregulated by 0.59 mM and 5.9 mM THGP (1.13 ± 0.17 and 0.84 ± 0.10) in the presence of HPX-XOD (Fig. 9B). Furthermore, at the protein level as well as the gene level, the index of IL6 production was increased in the oxidative stress treated group (2.24 ± 0.20) compared to the control (1.00 ± 0.10) but was significantly reduced by the 5.9 mM THGP treatment (1.35 ± 0.31) (Fig. 10). IL6 is produced at the site of inflammation, is involved in the production of acute-phase proteins and causes acute inflammation that leads to chronic inflammation^44^. IL6 is also known as a rheumatic factor, and anti-IL6 receptor antibody therapy has attracted attention as a potential technique for alleviating rheumatism^45,46^. In addition, elevated expression levels of the NR4A2 gene reportedly lead to increased expression of pro-inflammatory genes (IL8) and might play a role in the pathogenesis of rheumatoid arthritis^47^. Because repagermanium has also been reported to suppress chronic rheumatoid arthritis^48^, suppression of IL6 and NR4A2 gene expression by THGP might be a possible mechanism responsible for the relief of inflammation. In addition, the expression level of the chemokine CXCL2 gene was upregulated by HPX-XOD (4.69 ± 1.27) and downregulated by 0.59 mM and 5.9 mM THGP (2.24 ± 0.32 and 0.95 ± 0.05) in the presence of HPX-XOD (Fig. 9C). CXCL2 is a well-known chemokine involved in the recruitment of neutrophils and the inflammatory response^49,50^ that is produced by monocytes, macrophages, endothelial cells, fibroblasts, neural tissue, and a variety of tumor cells^51^. It has been reported that CXCL2 gene expression is correlated with the amount of ROS and IL6 mRNA as well as cell death^52,53^. Because the levels of intercellular ROS induced by HPX-XOD were not reduced by THGP (Fig. 7C), THGP might suppress the IL6 and CXCL2 genes or protein downstream of ROS in the inflammatory pathway, which might result in reductions in inflammatory signaling and cell death.

## Conclusions

In this study, we showed the intracellular localization of THGP and its preventive effect against ROS-induced cell death. THGP was incorporated into the cells and was more concentrated in the nucleus than in the cytoplasm of NHDFs. The present study also provided the first visual demonstration of the incorporation of THGP and/or germanium into cells. The results also showed that the incorporated THGP suppressed cell death due to oxidative stress in a dose-dependent manner. It has become clear that the suppressive effect of THGP on cell death is caused by a mechanism that differs from that of general antioxidants, which involve the scavenging of ROS. The mechanism might be caused by controlling genes related to cell death, such as NR4A2, and to inflammation, such as IL-6 and CXCL2. Unfortunately, the mechanism responsible for the effect of THGP on gene and protein expression remains unclear. Further experimental work is necessary to elucidate the mechanism underlying the suppressive effect of THGP on cell death, and this information might be obtained by examining the changes downstream of ROS in inflammatory pathways, such as the NFĸB signal transduction pathway, and cell death induction by NR4A2.

## Materials and Methods

### Reagents

Repagermanium (lot. 006316 A, purity > 99.9%), the polymer of THGP, was produced at the Hakodate plant of the Asai Germanium Research Institute Co., Ltd. (Hokkaido, Japan), and Bis(2-carboxyethylgermanium(IV) sesquioxide) was purchased from Sigma-Aldrich (Missouri, USA). Potassium bromide (KBr) and D_2_O were purchased from Kanto Chemical, Co., Inc (Tokyo, Japan). NHDFs and a Fibroblast Growth Medium 2 Kit were purchased from Takara Bio (Shiga, Japan). Xanthine oxidase (XOD from buttermilk) was purchased from Oriental Yeast Co., Ltd. (Tokyo, Japan). Hypoxanthine (HPX), hydrogen peroxide and 4% paraformaldehyde phosphate buffer solution were purchased from Wako Pure Chemical Industrial Co., Ltd. (Osaka, Japan). Cellstain-PI solution and Hoechst 33342 solution were purchased from Dojindo Laboratories Co., (Kumamoto, Japan). A complete tablets *EASY* pack was purchased from Roche Diagnostics GmbH (Mannheim, Germany). NR4A2 and β-actin antibodies were purchased from Santa Cruz Biotechnology (California, USA) and Abcam (Cambridge, England). Secondary antibodies, Goat Anti-Mouse IgG H&L (HRP) and Donkey Anti-Goat IgG H&L (HRP) were purchased from Abcam. All reagents used in this study were of special or molecular biological research grade.

### Measurement of differences in the material structure of Asai manufacturing and commercial repagermanium

We measured the infrared absorption spectra of repagermanium via the KBr method using Fourier Transform Infrared Spectrometer (Jasco Corporation, Tokyo, Japan) to investigate the structural differences between repagermanium manufactured by Asai and commercial repagermanium. A tablet was formed by adding 2 mg of pulverized crystals and 0.2 g of KBr to a press. Measurements were performed according to the manufacturer’s instructions, and the measuring wavenumber range was set to 4600–400 cm^−1^. Furthermore, we used a 300-MHz Varian Gemini 2000 spectrometer (Agilent Technologies, Santa Clara, CA, USA) to analyze differences in the molecular structure of the material. THGP was prepared with D_2_O at a concentration of 20 mM. Measurements were acquired at 30 °C, and 16 to 64 transients were used for the ^1^H analyses. Chemical shifts were expressed in ppm relative to the H resonance of HOD in the solvent, which was offset by 4.80 ppm.

### Evaluation of the incorporation of THGP into NHDFs

#### Imaging study

Silicon wafers were cut into 8-mm × 8-mm square pieces and sterilized at 160 °C for 3 h. The wafers were then placed on a 35-mm sterilized dish for cell culturing. NHDFs were seeded into the dish at 2.0 × 10^5^ cells/dish with 2 ml of culture medium. The cells were cultured at 37 °C under 5% CO_2_ for one day. The culture medium was then replaced by fresh culture medium with or without 10 mM THGP, and the cells were cultured for another day. After the medium was discarded, the cells on the silicon wafers were washed with physiological saline solution, and the washing solution was discarded. A drop of cold acetone (-80 °C) was then added to the cells on the wafers, and the cells were dehydrated for fixation under flowing nitrogen gas. After the cells were dry, a few drops of LR white resin (London Resin Company Ltd., Berkshire, England) with an accelerating reagent (5 µl/ml resin) were added to the cells. The resin solution was spread by a stream of nitrogen gas, and the resin caused the cells to harden for a few hours. For the imaging studies, including the isotope microscopy analysis, gold was deposited onto the cells on the silicon wafer.

The Hokudai isotope microscope system (CAMECA IMS 1270 and SCAPS ion imager at Hokkaido University) was employed to determine the distribution of ^74^Ge in NHDFs^18,19^. The mass spectra of metallic Ge ingot (purity of 99.999%; purified at Asai Germanium Research Institute Co., Ltd.) were obtained to identify the peaks corresponding to pure Ge. A Cs^+^ primary beam of 15 keV was homogeneously irradiated on the sample surface, which was approximately 80 µm in diameter, with a beam current of 10 nA. The instrument was tuned to avoid the interferences shown in Fig. 1 using a narrow exit slit. Negative secondary ions of ^74^Ge- sputtered from the sample surface were detected using a two-dimensional ion detector and visualized on a computer.

#### Determination of THGP

The incorporation of THGP into NHDFs was evaluated by liquid chromatography mass spectrometry (LC-MS/MS). Specifically, we measured the amount of THGP incorporated into NHDFs after culture with THGP by LC-MS/MS^54^. NHDFs were seeded into a six-well plate (TPP tissue culture plates, Sigma-Aldrich) at 2.0 × 10^5^ cells/well with 2 ml of culture medium. The cells were cultured for 24 h, and the medium was exchanged for a medium containing THGP. The final concentrations of THGP in the wells (n = 4) were 0, 1 and 10 mM THGP, and the cells were cultured for one day. The cells in each well cultured with each THGP concentration were collected, and 4.3 × 10^5^ viable cells in each well were counted via trypan blue staining. The medium was removed, and the cells were washed once with 1 ml of physiological saline solution, scraped out of the well and collected with 0.5 ml of ultra-purified water (UPW). The residues were transferred using another 0.5 ml of UPW. Ten microliters of an internal standard of 5 µg/ml of deuterium THGPNa ((HO)_3_GeCH_2_CD_2_COONa) was added to each sample (100 µl) and then converted to chloride salt using 200 µl of concentrated HCl for 5 min. After incubation, 2 ml of chloroform was added, and the samples were incubated with shaking for 1 h. The mixtures were centrifuged at 2,000 g for 10 min. The supernatant was discarded, the organic lower phase was transferred into another tube, and the solvent was evaporated. Finally, the sample was redissolved in 50 µl of pure water, and an aliquot was injected into the LC-MS/MS system. The LC conditions were as follows: a Nexera System (Shimadzu, Japan) with a Shodex DE413–2D column (150 mm × 2.0 mm) was used; the mobile phase was H_2_O/methanol/acetic acid (95:5:0.1, v/v/v); the flow rate was 0.3 ml/min; the column temperature was 50 °C; and the total analysis time was 5 min. The MS conditions were as follows: a TSQ Vantage EMR (Thermo Fischer Scientific, Massachusetts, USA) was used for tandem mass analysis; the spray voltage was 3000 V; the vaporizer temperature was 600 °C; the sheath gas pressure was 80 Arb; the AUX gas pressure was 20 Arb; the capillary temperature was 150 °C; and the declustering voltage was 0 V.

### Protective effect of THGP against oxidative damage in NHDFs

#### MTS-based measurement of cell proliferation under oxidative stress generated with hypoxanthine and xanthine oxidase

NHDFs were seeded in a 96-well plate (TPP tissue culture plates, Sigma-Aldrich) at 1.5 × 10^4^ cells/well and cultured under the conditions recommended by Takara Bio. When the cells reached semi-confluence the next day, the medium was exchanged for fresh medium, and 20 µl of 100 U/l XOD, 60 µl of 1 mM HPX and 20 µl of THGP were added to obtain a total medium volume in each well of 200 µl. The final concentrations of THGP in the wells were 0, 5.9, 59, 590 and 5900 µM. A negative control without THGP and XOD (HPX-XOD(−)) was prepared. Eight replicates of each group were included. The plates were incubated under 5% CO_2_ at 37 °C for 1.5 h. After the oxidative stress reaction, the old medium was exchanged for 100 µl of fresh medium, and the viable cells were then measured by the MTS (3-(4,5-dimethylthiazol-2-yl)-5-(3-carboxymethoxyphenyl)-2-(4-sulfophenyl)-2H-tetrazolium, inner salt) method. A 20-µl aliquot of MTS reagent (CellTiter 96 Aqueous One Solution Cell Proliferation Assay, Promega, Wisconsin, USA) was added, and the solution was incubated under 5% CO_2_ at 37 °C for 1 h. The formazan absorption of each well at 490 nm was then measured using a microplate reader ARVO3 (Perkin Elmer, Massachusetts, USA).

#### Measurement of cell proliferation by MTS and ATP production under oxidative stress induced by direct hydrogen peroxide addition

The effect of THGP on cell proliferation was evaluated under oxidative stress conditions in which hydrogen peroxide was added directly to the cells. In addition, the inhibitory effect of THGP on cell death was compared with that of AA, which is a common antioxidant. The procedure used for cell seeding was the same as that described above. The day after cell seeding, the medium was replaced with fresh medium, and 20 μl of 5 mM hydrogen peroxide and 20 μl of THGP solution or 20 μl of AA solution were added. The total medium volume of each well was 200 µl. The final concentrations of THGP in the wells were 0, 5.9, 59, 590 and 5900 µM, and the final concentration of AA in the wells was 100 μM. Furthermore, to investigate the effects of THGP and AA in the absence of oxidative stress, the cells were exposure to 0.59 mM and 5.9 mM THGP and 0.1 mM AA in the absence of hydrogen peroxide. After incubation using the above-described procedure, the viable cells were evaluated by MTS assay, and the intracellular ATP amount was measured using the CellTiter-Glo® 2.0 Assay kit (Promega, Wisconsin, USA) according to the manufacturer’s recommended protocol.

#### PI/Hoechst staining-based evaluation of cell death under oxidative stress induced by direct hydrogen peroxide addition

The effect of THGP on cell death due to hydrogen peroxide was examined by PI/Hoechst staining. NHDFs were seeded in a 12-well plate (TPP tissue culture plates, Sigma-Aldrich) at 1.5 × 10^5^ cells/well. The day after, the medium was changed to fresh medium, and 100 μl of 5 mM hydrogen peroxide was added to each well. Then, 100 μl of 59 mM THGP was added to the THGP-treated group, and the total volume of each well was 1 ml. The final concentrations of THGP in the wells were 0 and 5.9 mM, and the final concentration of hydrogen peroxide in the wells was 0.5 mM. A negative control without THGP and hydrogen peroxide was prepared. After culturing for 24 h, the culture medium was discarded, the cells were washed with PBS, and 5 μl of Hoechst solution (1 mg/ml) and 5 μl of PI solution (1 mg/ml) were added to obtain a total medium volume of 500 μl. The plates were incubated under 5% CO_2_ at 37 °C for 30 min in a dark environment. After incubation, the solution was discarded, and the cells were washed with PBS. Then, 500 μl of 4% paraformaldehyde phosphate buffer solution was added, and the cells were incubated at room temperature for 20 min for fixation. Finally, the stained cells were washed and observed with an inverted microscope (Eclipse TS100) equipped with a DS-Fi3 digital camera (Nikon, Tokyo, Japan). Excitation wavelengths of 361–389 nm and 540–580 nm were applied for Hoechst and PI, respectively. In each case, eight microscopic fields of 2 mm^2^ were randomly photographed with a fluorescence microscope at 40 × magnification, and the cells that emitted fluorescence were counted. The number of PI-positive cells was divided by the number of Hoechst-positive cells. The images were detected by a fluorescence microscope at 100 × magnification.

### Effect of the addition of THGP on antioxidative enzyme activity and the intracellular ROS content in NHDFs under oxidative stress

#### Evaluation of the antioxidative enzyme activity of NHDFs

NHDFs were seeded into six-well plates at 2.0 × 10^5^ cells/well and cultured in 2 ml of culture medium under the conditions recommended by Takara Bio. The cells were subjected to oxidative stress using the same protocol described for the HPX-XOD oxidative stress experiment but at 10-fold volumes. Additionally, the effects of THGP in the absence of oxidative stress were examined. The catalase and SOD activities of the samples were measured using a DetectX Catalase Colorimetric Activity Kit (Arbor Assays, Michigan, USA) and a SOD Assay Kit WST (Dojindo Laboratories, Kumamoto, Japan), respectively. After the samples were subjected to oxidative stress for 1.5 h, the supernatant was discarded, and 500 µl of cold buffer from the kit for measuring catalase activity or 500 µl of phosphate-buffered saline (PBS) for measuring SOD activity was added. The cells were scraped from the wells and collected. The wells were washed with 500 µl of cold buffer solution or PBS, and the collected cells were homogenized with an AM-5 homogenizer (Tokken, Chiba, Japan) at 16,000 rpm for 3 min. The homogenates were centrifuged at 10,000 g and 4 °C for 15 min, and the supernatants of the cell lysates were collected and used for further analysis. The measurements were conducted following the protocol suggested by the manufacturer.

#### Evaluation of intracellular ROS amounts of NHDFs

NHDFs were seeded in a 96-well plate at 1.5 × 10^4^ cells/well and cultured under the conditions recommended by Takara Bio. One day after cell seeding, the medium was discarded, Amplite^TM^ ROS Green working solution (AAT Bioquest, California, USA) was added, and the cells were incubated for 1 h. The cells were subjected to oxidative stress using the protocol used for the above-described HPX-XOD oxidative stress experiment, but the total volume was 120 µl. THGP was added at concentrations of 0.59 and 5.9 mM, and AA was added to the cells at concentrations of 0.01, 0.1 and 1 mM. The amount of intracellular ROS in NHDFs was determined according to the manufacturer’s instructions.

### Effect of THGP addition on the gene expression profile of NHDFs under oxidative stress

#### Gene expression profiling of skin cells after the application of THGP

NHDFs were cultured (1.0 × 10^5^ cells/well/2 ml) in six-well plates and cultured under the conditions recommended by Takara Bio. Two days after cell seeding, the NHDFs were exposed to oxidative stress by supplementation with 10 U/l XOD-0.3 mM HPX. THGP was added to the wells at concentrations of 0, 0.59 and 5.9 mM. The cells were then cultured under 5% CO_2_ at 37 °C for 1.5 h and harvested. Total RNA from the cells was extracted using Isogen (Nippon Gene, Toyama, Japan) according to the manufacturer’s instructions. Equal amounts of total RNA extracted from each of the six batches of cells were pooled, and their quality and quantity were evaluated by measuring the absorbance at a wavelength of 260/280 nm. The samples were labeled with the 3’IVT PLUS Reagent Kit (Affymetrix, California, USA) and hybridized with GeneChip Human Genome U133A (Affymetrix) according to the manufacturer’s recommended protocol. The hybridized chips were washed using GeneChip Fluidics Station 450 (Affymetrix) and scanned using a GeneChip Scanner 3000 7 G (Affymetrix). The data were normalized (robust multiarray analysis; RMA) and analyzed using Array Star software (version 4.0.2, DNASTAR Inc., Wisconsin, USA). Functional categorizing and clustering of the genes whose expression levels were altered by oxidative stress and/or THGP were performed using the Database for Annotation, Visualization and Integrated Discovery (DAVID) version 6.8 tools (http://david.abcc.ncifcrf.gov/)^55,56^.

#### Study of the expression of genes related to cell death and inflammation by quantitative polymerase chain reaction (qPCR)

The mRNA levels of genes encoding NR2A2 (NM_006186.3), IL6 (NM_000600.4) and CXCL2 (M57731) in the cells were determined by qPCR. The housekeeping gene RPS18 (NM_022551.2) was also examined as a reference gene^57,58^. cDNA was synthesized from the total RNA (as with the GeneChip experiment) by Super Script III reverse transcriptase (Invitrogen, California, USA). The cDNA samples were amplified with the primer set for each gene and SYBR Premix Ex Taq II (Tli RNase H Plus, Takara Bio) using a LightCycler 96 system (Roche Diagnostics GmbH). The primers used in this study were the following: NR4A2f, ctaacctgcaggcagaacctgaa; NR4A2r, acatttgtctgaactgcaacaacca; IL6f, aagccagagctgtgagatgagta; IL6r, tgtcctgcagccactggttc; RPS18f, tttgcgagtactcaacaccaacatc; RPS18r, gagcatatcttcggcccacac; CXCL2f, gcttgtctcaaccccgcatc; and CXCL2r, tggcctctgcagctgtgtct. The temperature program for the reaction was as follows: 95 °C for 30 s followed by 50 cycles of 95 °C for 5 s and 60 °C for 30 s.

### Evaluation of the production of IL6 of NHDF by Enzyme-Linked Immuno Sorbent Assay (ELISA)

The effect of THGP on the production of IL6 was evaluated under oxidative stress conditions, which were induced by the direct addition of hydrogen peroxide to the cells. The procedure used for the cell seeding and oxidative stress treatment was the same as that described in “*Measurement of cell proliferation by MTS and ATP production under oxidative stress induced by direct hydrogen peroxide addition*”. The experimental group consisted of 3 groups: untreated group, oxidative stress treated group and oxidative stress-THGP treated group. The THGP treatment was only performed at a final concentration of 5.9 mM. After the oxidative stress treatment, the culture supernatants were collected and used as a sample for measuring the production of IL6. IL6 was measured using a Human IL-6 ELISA Kit (Diaclone, Besançon, France) according to the manufacturer’s instructions. Viable cells were evaluated via a MTS assay, and the amount of IL6 culture supernatant was corrected by the viable cell rate.

### Statistical analysis

The results are presented as the means ± standard errors of the means (SEM). Outliers were statistically excluded by the Smirnov-Grabs test. The statistical analyses were performed using analysis of variance (ANOVA) followed by Dunnett’s tests and Steel methods for multiple comparison tests. The statistical significance of all the experiments was defined as p < 0.05.

## Supplementary information

Supplementary Figure S1, Supplementary Figure S2, Supplementary Figure S3
