# Supplementary material for: Organogermanium suppresses cell death due to oxidative stress in normal human dermal fibroblasts
Source: Sci Rep. 2019 Sep 20;9:13637. doi: 10.1038/s41598-019-49883-7 (PMC6754400; doi:10.1038/s41598-019-49883-7)

**Organogermanium suppresses cell death due to oxidative stress in normal human dermal fibroblasts**

**Tomoya Takeda^1,7^*, Sota Doiyama^1^, Junya Azumi^1^, Yasuhiro Shimada^1^, Yoshihiko Tokuji^2^, Hiroaki Yamaguchi^3,4^, Kosuke Nagata^5^, Naoya Sakamoto^6^, Hisashi Aso^7^ and Takashi Nakamura^1^**

1 Asai Germanium Research Institute Co., Ltd., 3-131, Suzuranoka, Hakodate, Hokkaido 042-0958, Japan

2 Department of Life and Food Sciences, Obihiro University of Agriculture and Veterinary Medicine, Nishi 2 Sen, Inada, Obihiro, Hokkaido 080-8555, Japan

3 Department of Pharmaceutical Sciences, Tohoku University Hospital, 1-1, Seiryo, Aoba, Sendai, Miyagi 980-8574, Japan

4 Yamagata University Graduate School of Medical Science/Department of Pharmacy, Yamagata University Hospital, 2-2-2, Iidanishi, Yamagata, Yamagata 990-9585, Japan

5 Department of Natural History Sciences, Hokkaido University, kita10jonishi, Kita, Sapporo, Hokkaido 060-0810, Japan

6 Isotope Imaging Laboratory, Creative Research Institution, Hokkaido University, kita10jonishi, Kita, Sapporo, Hokkaido 060-0810, Japan

7 Graduate School of Agricultural Science, Faculty of Agriculture, Tohoku University, 468-1, Aramaki aza, Aoba, Sendai, Miyagi 980-8578 Japan

Correspondence and requests for materials should be addressed to T.T. (e-mail: tomo.t621@asai-ge.co.jp)

**Supplementary Material**

Evaluation of the cellular NR4A2 protein expression of NHDF by western blotting (WB)

NHDFs were seeded in a 6-well plate at 4.5 × 10^5^ cells/well and cultured for a day. The medium was changed to fresh medium, and 200 μl of 5 mM hydrogen peroxide was added to each well at the final volume of 2 ml without the 5.9 mM THGP treatment. After culturing for 1.5 h, the culture medium was discarded and the cells were lysed with RIPA lysis buffer with protease inhibitor cocktail (Roche Diagnostics GmbH, Mannheim, Germany) on ice. The cell lysates were freeze thawed once to completely lyse the cells and centrifuged at 9,600 g and 4 °C for 10 min. The supernatants of the cell lysates were collected and their protein concentrations were measured using BIO-RAD Protein Assay (BIO-RAD, California, USA). Equal amount of proteins (7.5 μg) underwent 12% SDS-PAGE and then were transferred to Clear Blot Membrane-P plus membranes (ATTO corporation, Tokyo, Japan). The membranes were blocked in TBST containing 5% skim milk for 1 h at room temperature and then incubated with primary antibody diluted in TBST with skin milk (NR4A2; 1:100, β-actin; 1:5000) at 4 °C overnight. After three times washings with TBST, the membranes were incubated with appropriate horseradish peroxidase-conjugated secondary antibodies (NR4A2; 1:2500, β-actin; 1:5000) for 1 h at room temperature. The immunoreactive bands were developed with the Clarity ^TM^ Western ECL Substrate (BIO-RAD). β-actin was used as internal control. The NR4A2 and β-actin samples derive from the same gels/blots of the experiment were processed in parallel. The relative levels of proteins were analyzed with Image LabTM software (BIO-RAD). Background was acquired and subtracted from density volumes for NR4A2 and β-actin. Subsequently, NR4A2 densities were normalized to β-actin, and subsequently to nontreatment controls.

**Supplementary Data**

Supplementary Figure S1. Comparison of effects of Asai manufacturing and commercial THGP on the viability and intracellular ATP of NHDFs exposed to oxidative stress for 1.5 h.

Bis(2-carboxyethylgermanium(IV) sesquioxide) is referred to as commercial repagemanium. (A) NHDFs were cultured with or without THGP at a concentration of 5.9 mM, and oxidative stress was induced by the addition of 10 U/l XOD and 0.3 mM HPX. (B and C) NHDFs were cultured with or without THGP. THGP was added at a concentration of 0 or 5.9 mM. Oxidative stress was induced by the addition of 5 mM H_2_O_2_. The data are shown as the means, and the bars indicate the SEMs (n = 6 or 8). The asterisks indicate significant differences at p < 0.05. (A) Viable cells after exposure to HPX-XOD stress. (B) Viable cells after exposure to H_2_O_2_ stress. (C) Intracellular ATP levels after exposure to H_2_O_2_ stress.


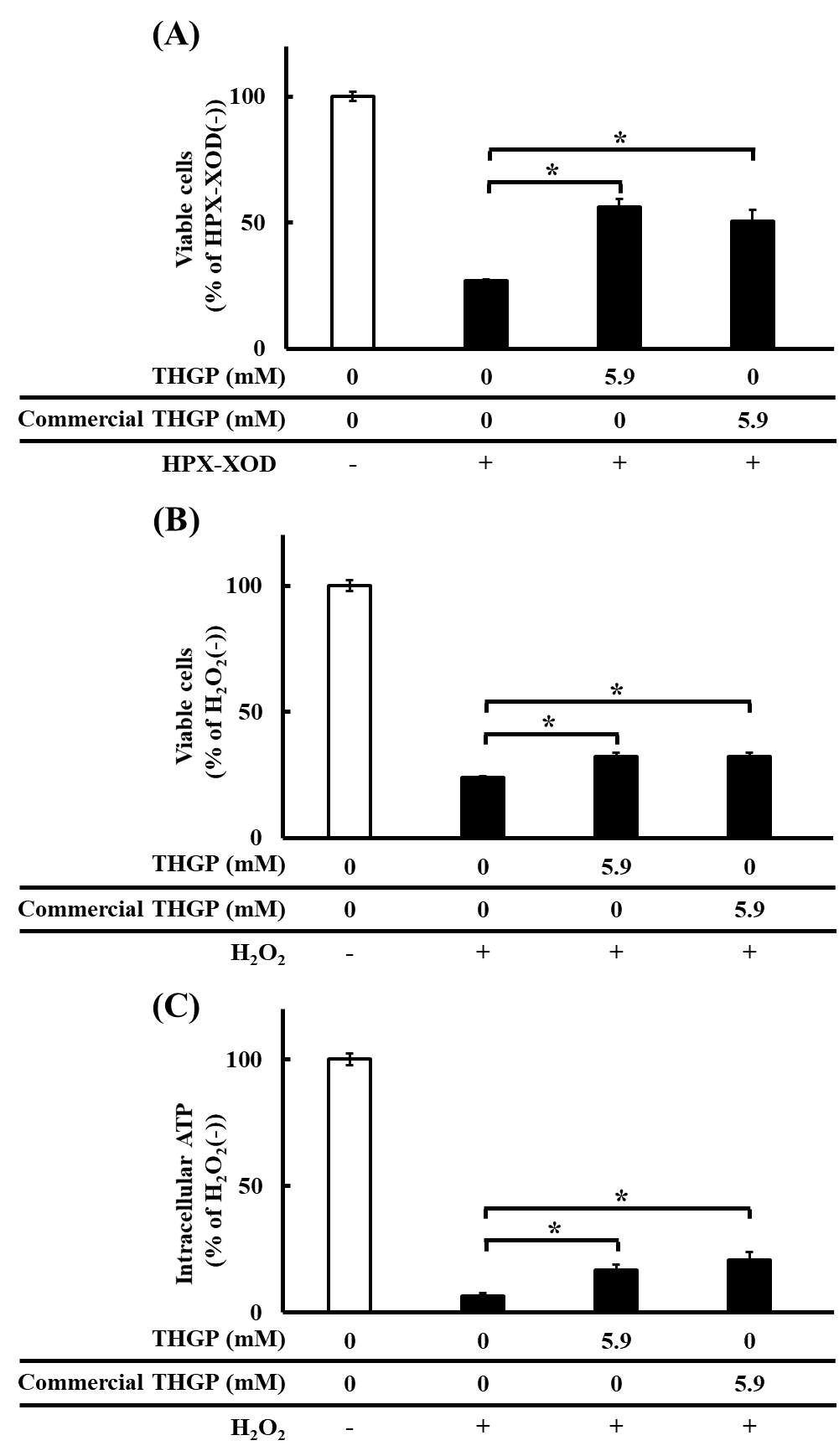
Supplementary Figure S1.

Supplementary Figure S2. Comparison of the effects of Asai manufacturing and commercial THGP on intracellular ROS in NHDFs.

Bis(2-carboxyethylgermanium(IV) sesquioxide) is referred to as commercial repagemanium. NHDFs were cultured with THGP at a concentration of 0 or 5.9 mM or ascorbic acid (AA) at 0.1 mM. Oxidative stress was induced by the addition of 10 U/l XOD and 0.3 mM HPX. The data are shown as the means, and the bars indicate the SEMs (n = 6). The asterisk indicates significant differences at p < 0.05.


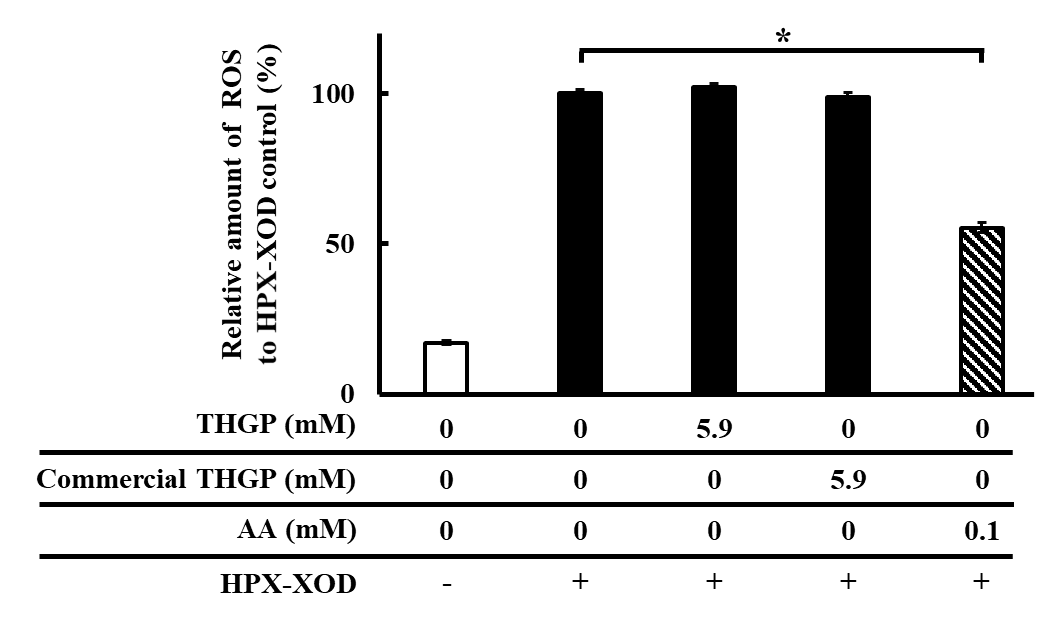
Supplementary Figure S2.

Supplementary Figure S3. Evaluation of the cellular NR4A2 protein expression of NHDF

NHDFs were cultured with THGP at a concentration of 0 or 5.9 mM. Oxidative stress was induced by the addition of 0.5 mM H_2_O_2_. WB showed the protein levels of NR4A2 and β-actin. The NR4A2 and β-actin samples derive from the same gels/blots of the experiment were processed in parallel. The results for three bands in each group are shown. The data are shown as the means, and the bars indicate the SEMs (n = 3).


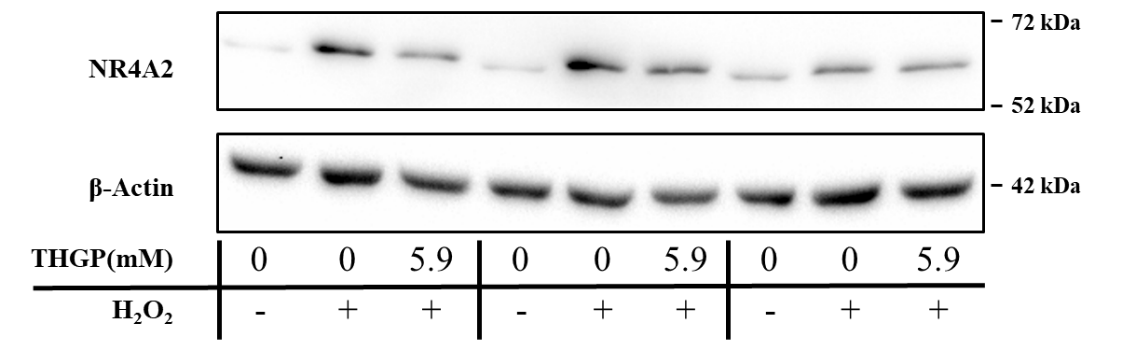
Supplementary Figure S3.


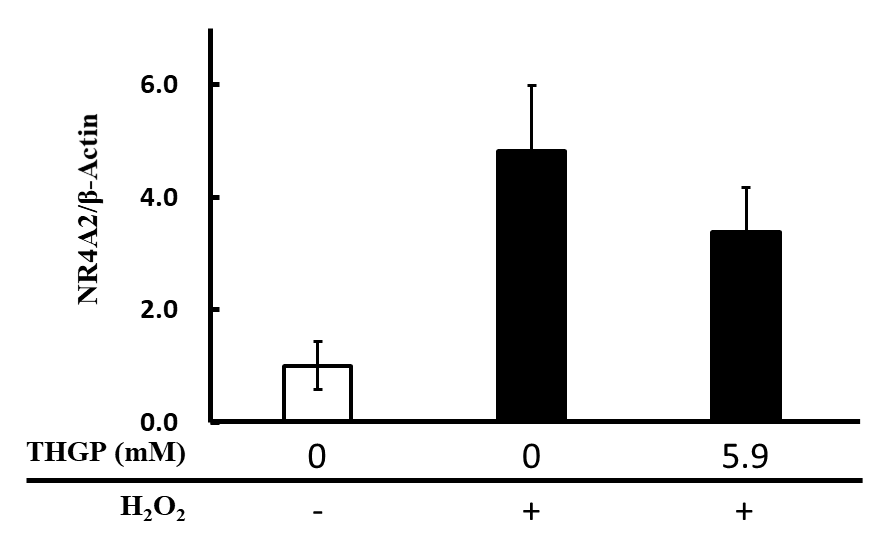

Supplement: Supplementary file 1 — Supplementary Figure S1, Supplementary Figure S2, Supplementary Figure S3 [file 41598_2019_49883_MOESM1_ESM.docx]
